# Molecular Interplay between the Dimer Interface and the Substrate-Binding Site of Human Peptidylarginine Deiminase 4

**DOI:** 10.1038/srep42662

**Published:** 2017-02-17

**Authors:** Chien-Yun Lee, Chu-Cheng Lin, Yi-Liang Liu, Guang-Yaw Liu, Jyung-Hurng Liu, Hui-Chih Hung

**Affiliations:** 1 Department of Life Sciences, National Chung Hsing University, Taichung, Taiwan; 2 Graduate Institute of Biotechnology, National Chung Hsing University, Taichung, Taiwan; 3 Molecular and Biological Agricultural Sciences Program, Taiwan International Graduate Program, Academia Sinica, Taipei, Taiwan; 4 Institute of Biochemistry, Microbiology and Immunology, Chung Shan Medical University, Taichung, Taiwan; 5 Division of Allergy, Immunology and Rheumatology, Chung Shan Medical University Hospital, Taichung, Taiwan; 6 Institute of Genomics and Bioinformatics, National Chung Hsing University, Taichung, Taiwan

## Abstract

Our previous studies suggest that the fully active form of Peptidylarginine deiminase 4 (PAD4) should be a dimer and not a monomer. This paper provides a plausible mechanism for the control of PAD4 catalysis by molecular interplay between its dimer-interface loop (I-loop) and its substrate-binding loop (S-loop). Mutagenesis studies revealed that two hydrophobic residues, W347 and V469, are critical for substrate binding at the active site; mutating these two residues led to a severe reduction in the catalytic activity. We also identified several hydrophobic amino acid residues (L6, L279 and V283) at the dimer interface. Ultracentrifugation analysis revealed that interruption of the hydrophobicity of this region decreases dimer formation and, consequently, enzyme activity. Molecular dynamic simulations and mutagenesis studies suggested that the dimer interface and the substrate-binding site of PAD4, which consist of the I-loop and the S-loop, respectively, are responsible for substrate binding and dimer stabilization. We identified five residues with crucial roles in PAD4 catalysis and dimerization: Y435 and R441 in the I-loop, D465 and V469 in the S-loop, and W548, which stabilizes the I-loop via van der Waals interactions with C434 and Y435. The molecular interplay between the S-loop and the I-loop is crucial for PAD4 catalysis.

Peptidylarginine deiminases (PADs) are a family of enzymes that catalyze the conversion of protein-arginine to protein-citrulline, referred to as protein deimination or citrullination. Protein citrullination is a naturally occurring post-translational modification; once protein substrates are citrullinated, the protein structures and protein-protein interactions may be altered due to the loss of charge in the structure^1^,^2^. Protein citrullination is important in physiological processes such as keratinization, myelin sheath stability in the brain, inflammation, and gene regulation^3^,^4^,^5^.

Five isoforms of PADs (PAD1-4, and PAD6) have been identified and are expressed in a tissue-specific manner^5^,^6^,^7^,^8^,^9^,^10^. PAD4 is the only isoform that contains a nuclear localization signal (NLS) and thus can be translocated into the nucleus^11^. PAD4 was first identified in human promyelocytic leukemia HL-60 cells, and its expression is induced when cells differentiate into granulocytes and monocytes^6^. PAD4-catalyzed histone hypercitrullination is required for antibacterial neutrophil extracellular trap (NET) formation, a pro-inflammatory response^12^. PAD4 also participates in chemokine function. Citrullinated CXCL8 has lower affinity for glycosaminoglycans, thus attenuating its ability to attract neutrophils to the peritoneum^13^. Moreover, citrullination of CXCL10 and CXCL11 reduces their signaling and chemoattraction of CXCR3, which may affect T cell activation^14^.

PAD4 functions as a transcriptional regulator by citrullinating its nuclear substrates, histone H2A, H3 and H4, to promote chromatin decondensation^6^. PAD4-catalyzed citrullination modulates gene expression by antagonizing histone Arg methylation, which converts methyl-Arg to citrulline and releases methylamine^15^,^16^. In addition, PAD4 functionally associates with other histone-modifying enzymes, such as histone deacetylase 1 and 2 (HDAC1 and HDAC2) and protein arginine N-methyltransferase 1 (PRMT1), to generate a repressive chromatin environment^17^,^18^,^19^,^20^. Specifically, PAD4 and HDAC1 co-repress the estrogen-regulated pS2 promoter^18^, and PAD4 and HDAC2 co-repress the promoters of p53 target genes such as p21, GADD45, and PUMA^19^.

Deregulation of PAD4 activity is correlated with several diseases^1^,^21^,^22^. The pathological role of PAD4 was first established in the autoimmune disease rheumatoid arthritis (RA). Citrullinated proteins are major autoantigens that accumulate in the joints of RA patients, thus leading to chronic pain and inflammation. The degree of inflammation in the RA synovium correlates with citrullination and PAD4 expression^23^, indicating an association between RA severity and PAD4 expression. In 2003, an RA-associated PAD4 haplotype consisting of 4 exonic single nucleotide polymorphisms (SNPs) was identified^24^, and the increase in PAD4 protein levels in the synovium of RA patients compared to healthy individuals is due to the increased stability of the PAD4 mRNA containing these SNPs.

PAD4 has recently emerged as an attractive drug target in the therapeutic treatment of cancer. Although its role in pathogenesis is not completely understood, deregulated PAD4 activity has been implicated in cancer progression because increased PAD4 expression is observed in various cancer cells and tissues^2^,^25^,^26^. Analogues of the PAD4 substrate benzoylarginine amide, F-amidine and Cl-amidine, are highly potent PAD4 inhibitors that effectively block the active site via their haloacetamidine groups^27^,^28^. Treatment with F-amidine, Cl-amidine, and their derivatives represses tumor growth in both cancer cell lines and mouse models^17^,^29^,^30^,^31^,^32^.

Crystal structures of PAD4 indicate that PAD4 is a homodimer in which one subunit contacts the other in a head-to-tail manner (Fig. 1A^33^). Five calcium ions are located in the N- and C-domains of each subunit (Fig. 1A^33^). As part of the elucidation of the relationship between structure and function in PAD4, we previously demonstrated that fully functional PAD4 should be in the dimeric form and that the non-catalytic calcium ions in the N-terminal domain are indispensable for the overall conformational stability of PAD4, which in turn is essential for full activation of the PAD4 enzyme^34^,^35^.

Although the human PAD2 and PAD4 isozymes have been reported to form a homodimer^33^,^36^, the crystal structure of PAD1 actually differs from that of PAD2 and PAD4. Studies of the solution and crystal structures of these isozymes have revealed that although PAD1 exists as an active monomer, PAD3 dimerizes to yield a structure similar to that of PAD2 and PAD4^37^. The flexible and substrate-accessible monomeric structure exclusive to PAD1 gives it a broader substrate specificity than the other isozymes^37^.

PAD4 has a total of two active sites, one in each subunit; these are located approximately 22 Å from the dimer interface, a long distance. However, monomeric PAD4 is much less active than dimeric PAD4^34^. In this paper, we demonstrate that the interplay between the dimer interface and the substrate-binding site of PAD4, which consist of the interface loop (I-loop) and the substrate-binding loop (S-loop), respectively (Fig. 1B), is responsible for substrate binding and dimer stabilization. The critical residues that participate in the molecular interplay between the dimer interface and the substrate-binding site are identified.

## Results and Discussion

### Kinetic and Biophysical Properties of Human PAD4 Substrate-Binding Site Mutants

**Figure 1:**
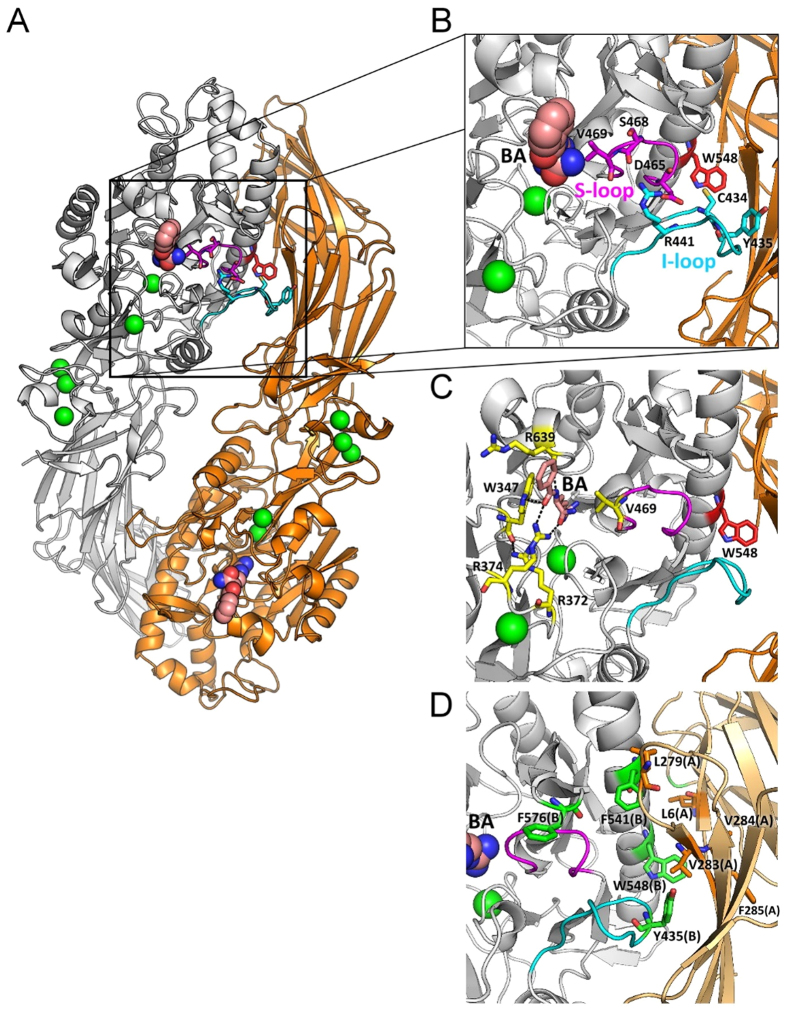
Structure of homodimeric PAD4 and the hydrophobic residues located at the substrate-binding site and the dimer interface. (**A**) Homodimer of human PAD4 (PDB ID: 1WDA). The bound calcium ions are indicated as green balls. The substrate analogue, benzoyl‐L‐arginine amide (BA), is shown as a sphere model with the carbon atoms in pink. The individual subunits are shown in gray and orange. (**B**) Detailed structure of the interface loop (I‐loop, cyan) and the substrate‐binding loop (S‐loop, magenta). The important residues on the loops are shown as stick models. (**C**) Active site of PAD4. The substrate‐binding residues are represented as yellow sticks, and the substrate BA is shown as pink sticks. The black dashed lines represent the polar contacts between the residues and the substrate. (**D**) Hydrophobic amino acid residues in the dimer interface of PAD4. The green and orange sticks indicate the residues associated with the different subunits.

The crystal structures of PAD4 complexed with an artificial substrate, benzoyl-L-arginine amide (BA), and its histone peptide substrates^33^,^38^ indicate that three arginyl residues, R372, R374 and R639, form hydrogen bonds with the amide oxygen and nitrogen of the substrate. In addition, two hydrophobic amino acid residues, W347 and V469, may regulate the stability of substrate binding (Fig. 1C). Here, we examine the significance of these active site amino acid residues in enzyme catalysis.

Mutation of R372 to alanine (R372A) or glutamine (R372Q) severely reduced the catalytic activity of PAD4; the *k*_cat_ values of R372A and R372Q were 0.07 s^−1^ and 0.12 s^−1^, respectively, approximately 1% of that of the wild-type (WT) enzyme (11.7 s^−1^). The *K*_m,BAEE_ values of these two mutants for the artificial substrate benzoyl-L-arginine ethyl ester (BAEE) were 3.7 mM and 2.3 mM, respectively, 5–7-fold higher than that of the WT enzyme (0.5 mM). Therefore, the overall catalytic efficiency (*k*_cat_/*K*_m_) of these two mutants was negligible compared with the WT enzyme (Table 1). The R372K enzyme exhibited no activity despite the conservation of the positive charge, suggesting that R372 is irreplaceable and critical for the catalytic activity of the PAD4 enzyme.

Mutation of the other two arginyl residues, R374 and R639, had a smaller impact on PAD4 catalysis. The *k*_cat_ values of R374A and R374Q were 7.8 s^−1^ and 3.7 s^−1^, respectively, approximately 30–60% of the activity of the WT enzyme (11.7 s^−1^). The *K*_m,BAEE_ values of these two mutants were 0.7 mM and 0.4 mM, respectively, similar to that of the WT enzyme (0.5 mM). The catalytic efficiency (*k*_cat_/*K*_m_) of R374A and R374Q was 40–70% that of the WT enzyme (Table 1). Similar results were observed with the R639A mutant. The *k*_cat_ value of R639A was 5.0 s^−1^, approximately 40% of the activity of the WT enzyme (11.7 s^−1^); its *K*_m,BAEE_ was 0.7 mM, similar to WT, and the *k*_cat_/*K*_m_ of R639A was 30% that of the WT enzyme (Table 1). These data indicate that R374 and R639 are important but not critical for PAD4 catalysis.

The kinetic data for the two hydrophobic amino acid residues W347 and V469 revealed that mutations at these sites severely impaired the catalytic activity of PAD4 (Table 1). The W347A mutant did not exhibit any detectable enzyme activity; W347F also did not exhibit activity, even though the hydrophobic property of this residue was conserved. Similar results were also observed with the V469 mutants. The V469A, V469L and V469T mutants displayed negligible enzyme activities; *k*_cat_ was 0.8 s^−1^, 0.1 s^−1^ and 0.04 s^−1^, respectively, and *K*_m,BAEE_ was 5.3 mM, 1.0 mM and 0.1 mM, respectively. The limited catalytic efficiency (*k*_cat_/*K*_m_) of the V469 mutants was mainly due to the reduced *k*_cat_ values (Table 1).

**Figure 2:**
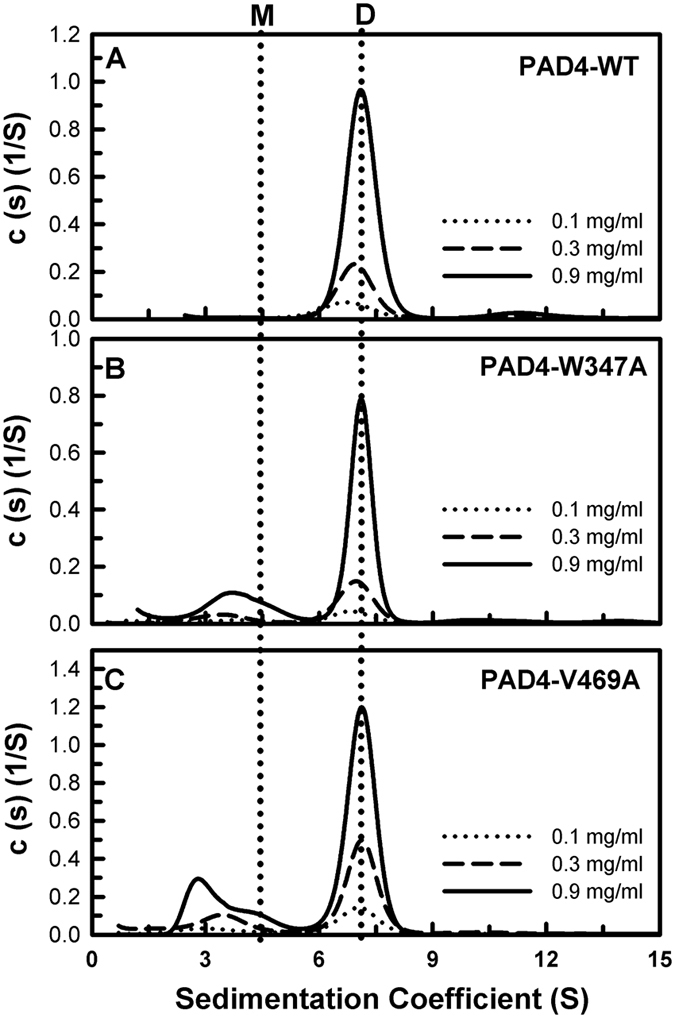
Continuous sedimentation coefficient distributions of PAD4 WT and the hydrophobic substrate-binding-site mutants. (**A**) WT. (**B**) W347A. (**C**) V469A. Three protein concentrations (0.1, 0.3, and 0.9 mg/ml) were analyzed as indicated in the figure. The sedimentation velocity data were globally fit using the SEDPHAT program to obtain the *K*_d_ (dissociation constant) of the PAD4 dimer (Table 1). M, monomer; D, dimer.

We examined the thermal stabilities and the quaternary structures of W347A and V469A. The melting temperatures (*T*_m_) of W347A and V469A were 47 °C and 50 °C, respectively (Table 1 and Figure S1), similar to that of PAD4 WT (51 °C). The PAD4 enzyme is in a stable dimeric form rather than in a monomeric form; the dimeric form is fully active, but the monomeric form is less active [34]. Thus, the W347A and V469A mutants with low enzymatic activity were examined by analytical ultracentrifugation to investigate possible changes in their quaternary structures. Self-association of W347A and V469A indicated that they still exist as stable dimers, albeit with higher dissociation constants (*K*_d_) of 1.02 μM and 2.13 μM, respectively (Table 1 and Fig. 2). Because W347A and V469A still exist as dimers, the limited catalytic ability of W347A and V469A is not due to dissociation of the enzyme dimers; therefore, W347 and V469 must play critical roles in the active site of PAD4.

### Kinetic and Biophysical Properties of the Human PAD4 Dimer-Interface Mutants

We continued to explore the hydrophobic amino acid residues that may be important for PAD4 catalysis. There are several hydrophobic amino acid residues in the dimer interface, including L6, L279, V283, V284 and F285 in the A subunit and F541, W548 and F576 in the B subunit (Fig. 1D). L6A, L279A, V283A, V284A, F285A and F541A displayed *k*_cat_ values that were 47–68% that of the WT enzyme, whereas the *K*_m,BAEE_ values for these mutants were similar to that of the WT enzyme (Table 2). W548A unexpectedly exhibited very limited enzyme activity, and its kinetic parameters could not be determined, whereas F576A retained most of the activity of the WT enzyme (Table 2).

**Figure 3:**
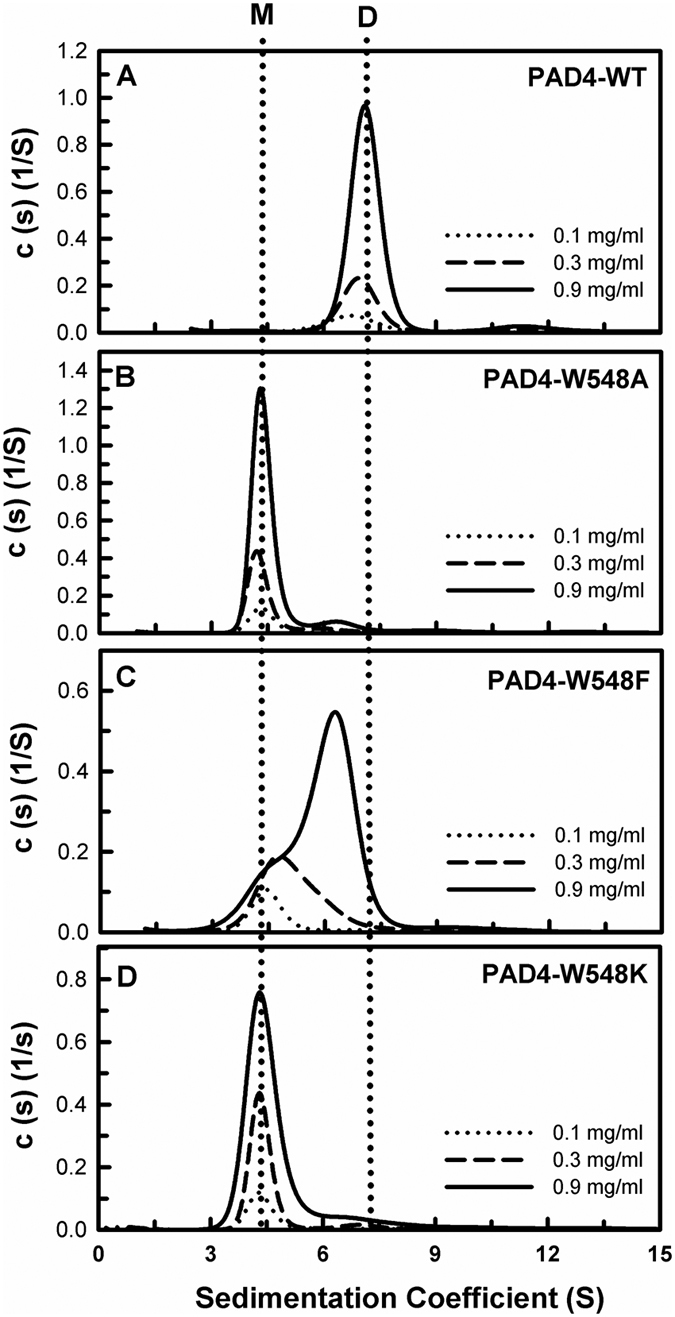
Continuous sedimentation coefficient distributions of PAD4 WT and the W548 series of dimer-interface mutants. (**A**) WT. (**B**) W548A. (**C**) W548F. (**D**) W548K. Three protein concentrations (0.1, 0.3, and 0.9 mg/ml) were analyzed as indicated in the figure. The sedimentation velocity data were globally fit using the SEDPHAT program to obtain the *K*_d_ of the PAD4 dimer (Table 2). M, monomer; D, dimer.

Because W548A exhibited almost no enzyme activity, the catalytic activities of the W548F and W548K enzymes were examined. The kinetic data demonstrated that W548F exhibited half of the enzyme activity of WT, whereas W548K displayed negligible enzyme activity; the *k*_cat_ values of W548F and W548K were 6.2 s^−1^ and 0.6 s^−1^, respectively (Table 2). The thermal stabilities and quaternary structures of the W548 series of mutants were also examined. The *T*_m_ values of W548A, W548F and W548K were 46 °C, 47 °C and 46 °C, respectively, 4–5 °C lower than that of PAD4 WT (51 °C; Table 2 and Figure S1). The W548A enzyme was present in monomeric form and displayed a *K*_d_ value of 88.7 μM, 887-fold larger than that of the WT enzyme; W548F existed in a monomer-dimer equilibrium with a *K*_d_ value of 12.9 μM, 129-fold larger than that of WT, and W548K was present in a monomeric form with a *K*_d_ value of 84.5 μM, 845-fold larger than that of WT (Table 2 and Fig. 3). These data indicate that the side chain of W548 is not only important for dimer stability but is also crucial for PAD4 catalysis because the other monomeric mutant of PAD4 remained active^34^.

The catalytic efficiencies of the L6A, L279A, V283A and V284A enzymes were 50% lower than that of the WT enzyme (Table 2). We further examined the effects of the side chains of these residues on enzyme activity using a series of mutants including L6I, L6D, L279I, L279D, V283I, V283D, V284I and V284D. The kinetic parameters of these mutants are shown in Table 2. For the L6 series of mutants, the *k*_cat_ of L6I further recovered to 10.9 s^−1^ (11.7 s^−1^ and 7.3 s^−1^ for WT and L6A, respectively); the *k*_cat_/*K*_m,BAEE_ value of L6I was 21.8 mM^−1^ s^−1^ (23.4 mM^−1^ s^−1^ and 12.2 mM^−1^ s^−1^ for WT and L6A, respectively). The L6D enzyme displayed a *k*_cat_ value of 4.5 s^−1^, 40% that of the WT enzyme (11.7 s^−1^ and 7.3 s^−1^ for WT and L6A, respectively), and its *k*_cat_/*K*_m,BAEE_ was 9.0 mM^−1^ s^−1^ (23.4 mM^−1^ s^−1^ and 12.2 mM^−1^ s^−1^ for WT and L6A, respectively). A similar pattern was observed for the L279, V283 and V284 mutants. In the L279 series of mutants, the *k*_cat_ of L279I significantly recovered to 11.2 s^−1^ (11.7 s^−1^ and 5.5 s^−1^ for WT and L279A, respectively), and its *k*_cat_/*K*_m,BAEE_ value was 28 mM^−1^ s^−1^ (23.4 mM^−1^ s^−1^ and 13.8 mM^−1^ s^−1^ for WT and L279A, respectively). The L279D enzyme displayed a *k*_cat_ value of 3.6 s^−1^, 30% that of the WT enzyme (11.7 s^−1^ and 5.5 s^−1^ for WT and L279A, respectively), and its *k*_cat_/*K*_m,BAEE_ was only 1.9 mM^−1^ s^−1^ (23.4 mM^−1^ s^−1^ and 13.8 mM^−1^ s^−1^ for WT and L279A, respectively). In the V283 series of mutants, the *k*_cat_ of V283T was 5.5 s^−1^, and the *k*_cat_/*K*_m,BAEE_ value of V283T was 6.3 s^−1^ mM^−1^. The *k*_cat_ of V283I was 9.5 s^−1^ (11.7 s^−1^ and 8.0 s^−1^ for WT and V283A, respectively), and its *k*_cat_/*K*_m,BAEE_ value was 19 s^−1^ mM^−1^ (23.4 mM^−1^ s^−1^ and 11.4 mM^−1^ s^−1^ for WT and V283A, respectively). The V283D enzyme did not exhibit any enzyme activity and was an inactive mutant enzyme. For the V284 series of mutants, the *k*_cat_ of V284I significantly recovered to 10.9 s^−1^ (11.7 s^−1^ and 5.5 s^−1^ for WT and V284A, respectively), and the *k*_cat_/*K*_m,BAEE_ value was 21.8 mM^−1^ s^−1^ (23.4 mM^−1^ s^−1^ and 3.7 mM^−1^ s^−1^ for WT and V284A, respectively). The V284D enzyme displayed a *k*_cat_ value of 2.9 s^−1^, 25% of that of the WT enzyme (11.7 s^−1^ and 5.5 s^−1^ for WT and V284A, respectively) and its *k*_cat_/*K*_m,BAEE_ was only 3.2 mM^−1^ s^−1^ (23.4 mM^−1^ s^−1^ and 3.7 mM^−1^ s^−1^ for WT and V284A, respectively).

**Figure 4:**
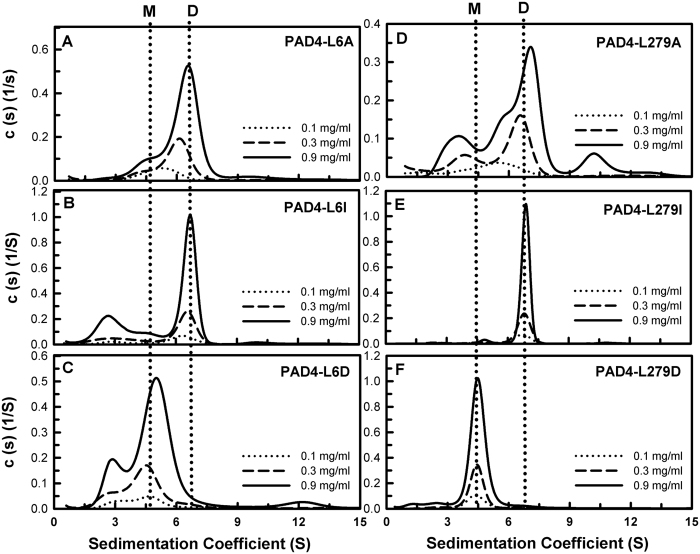
Continuous sedimentation coefficient distributions of the PAD4 L6 and L279 series of dimer-interface mutants. (**A**) L6A. (**B**) L6I. (**C**) L6D. (**D**) L279A. (**E**) L279I. (**F**) L279D. The *K*_d_ values of these mutants are shown in Table 2. M, monomer; D, dimer.

**Figure 5:**
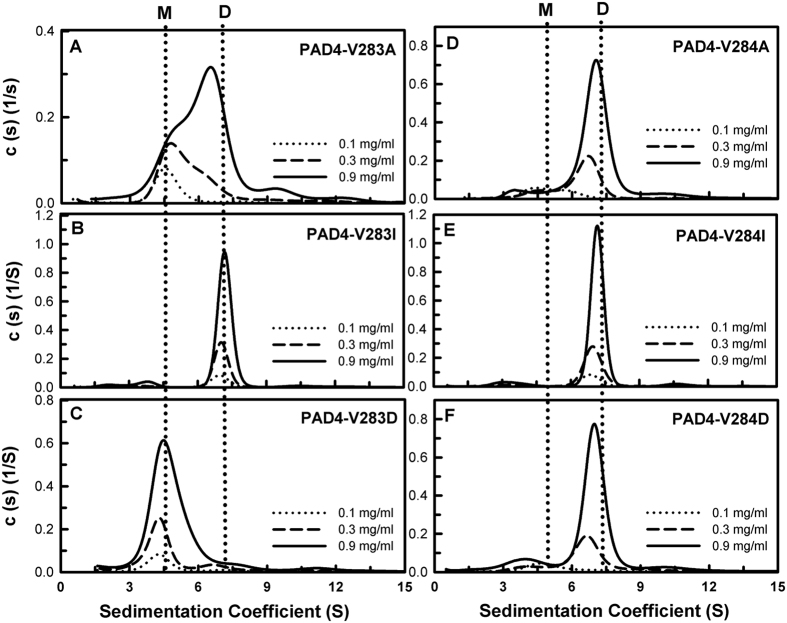
Continuous sedimentation coefficient distributions of the PAD4 V283 and V284 series of dimer-interface mutants. (**A**) V283A; (**B**) V283I; (**C**) V283D; (**D**) V284A; (**E**) V284I; (**F**) V284D. The *K*_d_ values of these mutants are shown in Table 2. M, monomer; D, dimer.

We also examined the thermal stabilities and quaternary structures of these hydrophobic amino acid mutants (Table 2). In the L6 series of mutants, the *T*_m_ values of L6A, L6I and L6D were 44 °C, 45 °C and 43 °C, respectively, 6–8 °C lower than that of PAD4 WT (51 °C; Figure S1). The L6A enzyme existed in a monomer-dimer equilibrium with a *K*_d_ value of 7.6 μM, 76-fold larger than that of WT; L6I was mainly present in a dimeric form with a *K*_d_ value of 2.13 μM, 21-fold larger than that of WT, and L6D was present in a monomeric form with a *K*_d_ value of 24.7 μM, 247-fold larger than that of WT (Table 2 and Fig. 4). Similar results were observed for the L279 and V283 series of mutants. For the L279 series of mutants, the *T*_m_ values of L279A, L279I and L279D were 46 °C, 48 °C and 45 °C, respectively, 3–6 °C lower than that of PAD4 WT (Table 2 and Figure S1). The L279A enzyme existed in a monomer-dimer equilibrium with a *K*_d_ value of 5.97 μM, 59.7-fold larger than that of WT; L279I was present as a stable dimer with a *K*_d_ value of 0.33 μM, similar to that of the WT enzyme, and L279D was present in a monomeric form with a *K*_d_ value of 65.3 μM, 653-fold larger than that of WT (Table 2 and Fig. 4). In the V283 series of mutants, the *T*_m_ values of V283A and V283D were 47 °C and 46 °C, respectively, 5 °C lower than that of PAD4 WT; however, V283I and V283T presented *T*_m_ values (54 °C and 54 °C, respectively) that were higher than that of the WT enzyme (Table 2 and Figure S1). The V283A and V283T enzymes existed in monomer-dimer equilibria with *K*_d_ values of 9.25 μM and 8.91 μM, respectively, approximately 90-fold larger than that of WT. V283I was present as a stable dimer with a *K*_d_ value of 0.18 μM, similar to that of WT, and V283D was present in monomeric form, with a *K*_d_ value of 46.7 μM, 467-fold larger than that of WT (Table 2 and Fig. 5).

In the V284 series of mutants, the *T*_m_ value of V284A and V284D was 47 °C, 4 °C lower than that of PAD4 WT, and V284I presented a *T*_m_ value of 52 °C, slightly higher than that of the WT enzyme (Table 2 and Figure S1). In contrast to the L6, L279 and V283 series of mutants, the L284 mutants were mainly present in the dimeric form; the *K*_d_ values of V284A, V284I and V284D were 2.4 μM, 0.18 μM and 2.59 μM, respectively (Table 2 and Fig. 5). Three Phe mutants, F285A, F541A and F576A, displayed protein stabilities similar to that of WT; the *T*_m_ values of these Phe mutants were approximately 47–48 °C, 3–4 °C lower than that of PAD4 WT. The F285A, F541A and F576A mutants were present as stable dimers; their *K*_d_ values were 0.75 μM, 0.25 μM and 0.12 μM, respectively (Table 2 and Figure S2). These data suggest that V284, F285, F541 and F576 are not important for dimer stability.

The kinetic and biophysical studies of the L6, L279, V283, V284 and W548 series of mutants reveal several interesting findings. First, substitution of L6, L279, V283 and V284 with another hydrophobic residue, Ile, preserved catalytic efficiency, thermal stability and dimeric structure, whereas substitution with the charged hydrophilic residue Asp resulted in the lowest catalytic activity among the respective series of mutants. Second, the observed changes in the thermal stability and quaternary structures of the L6, L279 and V283 series of mutants indicated that L6I, L279I and V283I were the most stable and displayed a dimeric structure nearly identical to that of WT; by contrast, L6D, L279D and V283D were the least stable and displayed a monomeric structure. These data suggest that although the side chains of L6, L279, V283 and V284 are not required for PAD4 catalysis and may be replaced with other hydrophobic residues, the hydrophobicity of these residues is required for protein stability and dimer formation. Third, W548 is unique among these hydrophobic amino acid residues because substitution of Trp by Ala resulted in an inactive enzyme and dissociation into monomers. Even when W548 was replaced with Phe, the catalytic efficiency of the mutant remained low, and the mutant was present in monomer-dimer equilibrium. Therefore, we suggest that the side chain of this Trp residue is not only required for dimer formation but is also crucial for the catalytic activity of PAD4. Next, molecular dynamic simulations of PAD4 WT and W548 mutants were performed.

### Molecular Dynamics (MD) Simulations of PAD4 WT and W548 Mutants

**Figure 6:**
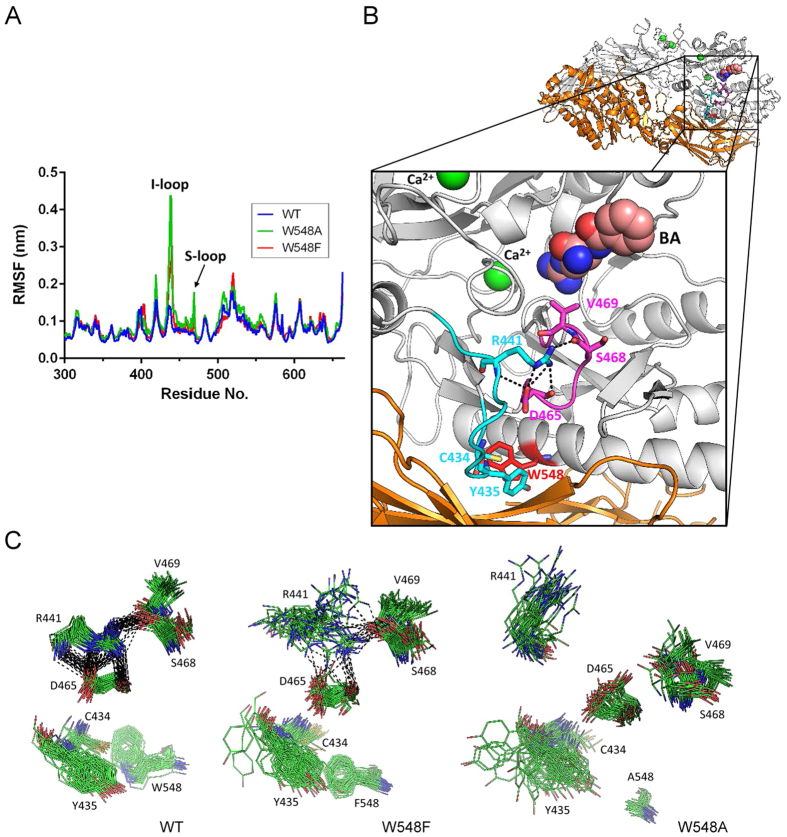
MD simulations of PAD4 WT and the W548 mutants. (**A**) RMSF for the Cα atoms in the C-terminal domain of the WT enzyme and the W548 mutants. The locations of the I-loop and the S-loop are labeled. (**B**) Structures of the I-loop (cyan) and the S-loop (magenta) in the PAD4 C-terminal domain. The PAD4 monomer after MD simulation is shown as a gray ribbon. To demonstrate the location of the loops in the dimer interface, we reconstituted the structure into a dimer in this panel; the other subunit is depicted as an orange ribbon. These important residues are highlighted as stick models and labeled. The bound calcium ion is depicted as a green ball. The substrate analogue BA is shown as a sphere model with the carbon atoms in pink. The possible polar contacts are represented by black dashed lines. (**C**) Frames of interaction between the residues in the I-loop and the S-loop during a 10-ns MD simulation. The black dashed lines represent the polar contacts between the two loops.

To investigate the structural consequences of W548 mutations, we conducted MD simulations of PAD4 WT and the W548A and W548F mutants. The potential energy and the root mean square deviation (RMSD) of the backbone atoms were calculated from the starting monomeric structures. As shown in Figure S3, the potential energies of the three MD systems decreased slightly and converged after 5 ns. The RMSD values of the WT and mutant structures also suggested that the three systems deviated from their starting structures to similar extents after 5 ns. To evaluate the structural flexibility of WT and the W548 mutants, we calculated the root mean square fluctuations (RMSF) for the Cα atoms from the last 10 ns of the simulation trajectory files. The RMSF values for the C-terminal domain of the WT and mutant structures are shown in Fig. 6A. The W548A and W548F mutants showed higher fluctuations in residues 435 to 470. A significant change in fluctuation was observed in the loop containing residues 433 to 443 (Fig. 6A). This loop, which is known as the interface loop (I-loop), is located at the dimer interface (Fig. 6B) and is important for PAD4 dimer formation^34^. The second notable loop with substantial fluctuations is located between residues 465 and 470 (Fig. 6A). We named this loop the ‘substrate-binding loop’ (S-loop, Fig. 6B) because it forms part of the substrate-binding pocket^38^. V469 is responsible for the interaction of the protein with the aliphatic portion of the arginine substrate. The conformation of the S-loop is associated with the I-loop because the side-chain nitrogen atoms of R441 can form hydrogen bonds with the backbone oxygen atoms of D465 and because the side-chain oxygen atom of D465 interacts with the side-chain and backbone nitrogen atoms of R441 (Fig. 6B). In fact, the conformations of the I-loop and the S-loop share similarity with those found in the crystal structure (Figure S4). In addition, the polar contacts between R441 and D465 are also found in the crystal structure.

Comparison of the 10-ns MD trajectories of the WT and W548 mutant structures (Fig. 6C) revealed that the conformations of the I-loop and the S-loop in the WT structure are quite stable. Obvious flexibility and losses of polar contacts were observed in the W548F structure. In the W548A structure, contact between the I-loop and the S-loop disappears, and the distance between the loops increases. We also observed that the conformational fluctuation of the Y435 side chain is related to the bulkiness of residue 548; in the WT structure, Y435 has a nearly ordered conformation, but the side-chain orientation of Y435 in the W548A mutant structure is random. We also noted that C434 bridges the interaction between W548 and Y435 in the simulated WT structure (Fig. 6B and C). These three residues form a compact cluster via numerous van der Waals contacts. Therefore, we hypothesize that the interaction between W548, C434 and Y435 may be important for the conformational stability of the I-loop.

### Kinetic and Biophysical Properties of the Human PAD4 I-Loop and S-Loop Mutants

**Figure 7:**
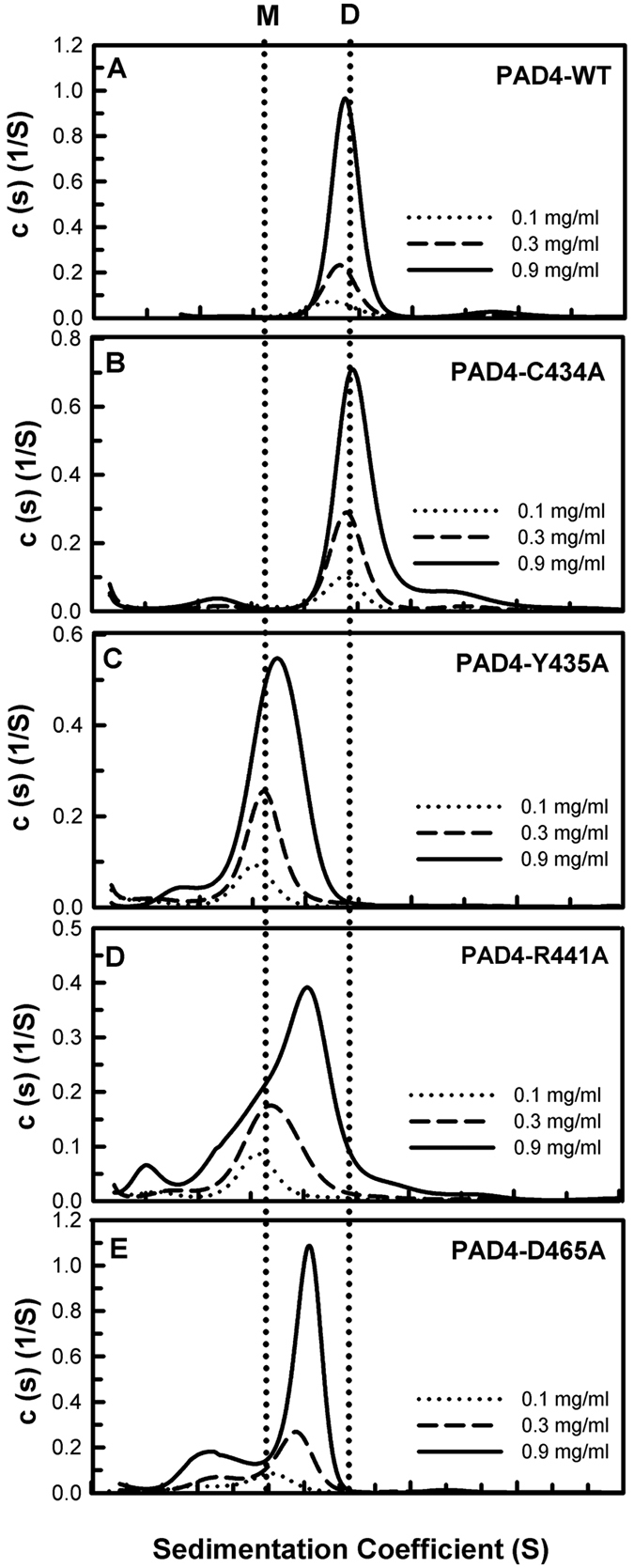
Continuous sedimentation coefficient distributions of the PAD4 I-loop and S-loop mutants. (**A**) WT. (**B**) C434A. (**C**) Y435A. (**D**) R441. (**E**) D465A. The *K*_d_ values of these mutants are shown in Table 3. M, monomer; D, dimer.

Compared to the WT structure, the simulated structure of W548A exhibited greater fluctuation; some polar contacts between R441, D465 and V469 disappeared, and the distance between Y435 and A548 increased substantially (Fig. 6C). Therefore, further mutagenesis of C434, Y435 and R441, all of which are located in the I-loop, and D465, which is located in the S-loop, was performed. The kinetic and biophysical properties of the C434A mutant were similar to those of the WT enzyme (Table 3); the *k*_cat_ and *k*_cat_/*K*_m,BAEE_ values of C434A were 8.0 s^−1^ (11.7 s^−1^ for WT) and 20 mM^−1^ s^−1^ (23.4 mM^−1^ s^−1^ for WT), respectively. The *T*_m_ of C434A was 50 °C (51 °C for WT), and the mutant was present as a stable dimer (Fig. 7B) with a *K*_d_ value of 0.36 μM (0.10 μM for WT). These results suggest that substitution of Ala at position 434 still permits stabilization of the I-loop. Y435 is important for dimerization^34^. The Y435A mutant was present as a monomer (*K*_d_: 22 μM; Fig. 7C) and displayed low enzyme activity (Table 3); its *k*_cat_ and *k*_cat_/*K*_m,BAEE_ values were 2.8 s^−1^ (11.7 s^−1^ for WT) and 0.9 mM^−1^ s^−1^ (23.4 mM^−1^ s^−1^ for WT), respectively. These data suggest that the aromatic side chain of Y435 is required to stabilize the conformation of the I-loop.

In the simulated structure of W548A, the molecular interactions between R441 and D465 nearly vanished (Fig. 6C). The R441A and D465A mutants displayed limited enzyme activity; the *k*_cat_ values were 2.3 s^−1^ and 3.5 s^−1^ (11.7 s^−1^ for WT), and the *k*_cat_/*K*_m,BAEE_ values were 1.4 mM^−1^ s^−1^ and 8.8 mM^−1^ s^−1^ (23.4 mM^−1^ s^−1^ for WT), respectively (Table 3). The *T*_m_ values of R441A and D465A were 47 °C and 46 °C, respectively, 4–5 °C lower than that of WT. In addition, both the R441A and D465A enzymes existed in monomer-dimer equilibrium with *K*_d_ values of 14.2 μM and 8.3 μM, respectively (Fig. 7D and E, respectively; Table 3). Thus, the molecular contacts between R441 and D465 play a vital role in the I-loop and the S-loop to maintain the correct geometry of the dimer interface and the substrate-binding site, respectively.

### Molecular Interplay between the I-Loop and the S-Loop

PAD4 is a dimer with two separate active sites in which all of the catalytic and substrate-binding residues are in the same subunit (Fig. 1C). We previously demonstrated that the fully active form of PAD4 should be a dimer rather than monomers^34^. This paper further reveals the interplay between the dimer interface and substrate-binding site of PAD4.

In the first part of this study, we identified several residues that are essential for the binding of PAD4 to its substrate. Kinetic studies revealed that a water-mediated hydrogen bond between R372 and the carbonyl oxygen of the arginine substrate is crucial and that R374 and R639 are also essential for binding to the substrate (Fig. 1C). In addition, both W347 and V469 are unexpectedly important for stabilizing the aliphatic portion of the arginine substrate. Therefore, mutation of these residues leads to a severe reduction in the catalytic activity of PAD4.

In the second part, we identified several hydrophobic amino acid residues at the dimer interface that affect catalytic activity and dimerization, particularly L6, L279 and V283 (Fig. 1D). Loss of hydrophobicity at these three residues impairs dimerization, thus reducing enzyme activity (Table 2).

Based on the MD simulations and mutagenesis studies, we suggest that the conformation of the I-loop (C434, Y435 and R441 in Fig. 6B) may regulate the enzymatic activity of PAD4 because the correct conformation of the S-loop (Fig. 6B; D465, S468 and V469 therein) depends on the stable conformation of the I-loop. Furthermore, dimerization of PAD4 is essential for its enzymatic function because dimerization can stabilize the conformation of the I-loop. Mutation of R441 or D465 disrupts the interaction between the I-loop and the S-loop; as the I-loop shifts away from the S-loop, it becomes flexible and impedes the dimerization of PAD4. Therefore, we suggest that R441 in the I-loop and D465 in the S-loop are involved in the interplay between the I-loop and the S-loop.

W548 is unique in the dimer interface; the replacement of this residue with Ala results in an inactive monomer, implying that W548 plays dual roles in catalysis and dimerization. MD simulations indicated that numerous van der Waals’ contacts between W548, C434 and Y435 result in a stable conformation of the I-loop (Fig. 6B and C). In the crystal structure of PAD4, the conformation of the I-loop is mainly stabilized through formation of the dimer (Figs 1D and S5). After dimerization, W548 interacts not only with C434 in the same subunit but also with residues V283 and Q286 in the neighboring subunit. C434 also interacts with V283 and Q286 in the neighboring subunit. In particular, Y435 flips away from C434 and forms intensive interactions with residues in the other subunit. It makes polar contacts with Y237 and E281 and van der Waals contacts with V200, H202 and L272 (Figure S5). Based on these findings, we hypothesize that W548 secures the conformation of the I-loop in a ‘dimerization-ready’ state in monomeric PAD4. Although this conformation is not precise enough to coordinate the S-loop in the catalysis process, it may facilitate the progress of dimerization. After dimerization, the dimer interface eases the duty of W548 and further guides the conformation of the I-loop into a ‘catalysis-ready’ state. Therefore, the substitution of W548 by Ala has several consequences. First, dimer formation is decreased due to the loss of stabilizing forces between the I-loop and residue 548; second, enzyme activity is reduced due to the loss of the molecular contacts between R441, D465 and V469. As the I-loop becomes flexible, the tendency to dimerize decreases; consequently, the S-loop becomes disordered, impairing the enzymatic activity of PAD4.

In summary, we provide a plausible mechanism for the control of PAD4 catalysis by the molecular interplay between the dimer-interface loop and the substrate-binding loop. Because PAD4 is considered a molecular target for therapeutic purposes, strategies that target the interactions between the S-loop and the I-loop may improve rational drug design.

## Experimental Procedures

### Site-Directed Mutagenesis

Site-directed mutagenesis to create mutant constructs of human PAD4 was performed using the Quik-Change mutagenesis kit (Agilent Technologies, Palo Alto, CA, USA). Wild-type (WT) DNA of human PAD4 was used as the template, and primers with the desired codon changes were used for PCR (polymerase chain reaction) with PfuUltra Fusion HS DNA polymerase (Agilent Technologies). The primers with the desired mutations ranged from 25 to 45 bases in length to ensure specific binding to the template DNA. After 28–30 temperature cycles, the mutated plasmids were treated with DpnI to digest the WT templates, and the plasmid DNAs with the desired mutations were then transformed into the XL-1 strain of *Escherichia coli*. DNA sequences with specific mutations were confirmed by sequencing. The sequences of the mutagenic primers used in this study are listed in Table S1.

### Expression and Purification of Recombinant Human PAD4

The human WT and mutant PAD4 genes were cloned into the pQE30 vector with an N-terminal 6xHis-tag. This ampicillin-resistant vector was used to transform the JM109 strain of *Escherichia coli*. Induction of PAD4 overexpression was induced with 1 mM isopropyl-1-thio-β-D-galactoside (IPTG) for 18–20 h at 25 °C. The overexpressed PAD4 proteins were purified using Ni-NTA agarose (Sigma, St. Louis, MO, USA). After mixing the lysate and resin, binding buffer (5 mM imidazole in 500 mM NaCl, 2 mM *β*-mercaptoethanol and 30 mM Tris-HCl, pH 7.6) and washing buffer (10 mM imidazole in 500 mM NaCl, 2 mM *β*-mercaptoethanol and 30 mM Tris-HCl, pH 7.6) were used to elute non-specifically bound proteins; the PAD4 protein was then eluted with elution buffer (250 mM imidazole, 500 mM NaCl, 2 mM *β*-mercaptoethanol and 30 mM Tris-HCl, pH 7.6). The purified PAD4 was dialyzed against dialysis buffer (500 mM NaCl, 2 mM *β*-mercaptoethanol, and 50 mM Tris-HCl, pH 7.6) and concentrated using a centrifugal filter device (Amicon Ultra-15, Merck Millipore, Billerica, MA, USA) with a 30-kDa molecular weight cutoff. The purity of the protein was verified by SDS-PAGE, and protein concentrations were estimated by the Bradford method^39^.

### Enzyme Assay of PAD4 and Analysis of Kinetic Data

The protocol used to continuously measure PAD4 enzyme activity coupled to a glutamate dehydrogenase-catalyzed reaction has been reported previously^40^. The reaction mixture for the spectrophotometric assay of PAD4 activity contained 10 mM benzoyl-L-arginine ethyl ester (BAEE) as the *in vitro* substrate for PAD4, 10 mM CaCl_2_, 2.5 mM dithiothreitol, 8.5 mM α-ketoglutarate (α-KG), 0.22 mM NADH and 8.4 U of glutamate dehydrogenase (GDH) in 100 mM Tris-HCl (pH 7.5) in a total volume of 1 ml and was incubated at 25 °C. The reaction was initiated by adding the appropriate amount of enzyme to the reaction mixture; the decrease in absorbance at 340 nm due to NADH oxidation was continuously monitored using a Perkin-Elmer LAMBDA-25 spectrophotometer. One enzyme unit was defined as the amount of enzyme that catalyzed the oxidation of 1 μmol of NADH per min. An extinction coefficient of 6220 cm^−1^M^−1^ for NADH at 340 nm was employed in the calculations. The *K*_m_ value of BAEE was determined by varying its concentration near its *K*_m_ value, and the *k*_cat_ value of PAD4 was calculated using Equation 1:


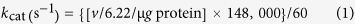


where *v* is ΔA_340_/min, 6.22 is the absorption coefficient of 1 mM NAD(P)H, 148,000 is the molecular weight of the human PAD4 dimer, and 60 indicates sixty seconds. The data were graphically analyzed with Sigma Plot 8.0 (Jandel, San Rafael, CA, USA).

### Analytical Ultracentrifugation

The quaternary structure of PAD4 was analyzed using an Optima XL-A analytical ultracentrifuge (Beckman Coulter, CA, USA). The concentrations of the PAD4 enzyme in the sedimentation velocity (SV) experiments were fixed at 0.1, 0.3, and 0.9 mg/ml. The sample cell was loaded with 370 μl of sample, and the reference cell was loaded with 400 μl of 30 mM Tris-HCl and 250 mM NaCl (pH 7.6). After the protein samples were loaded, the cells were transferred to an An-50 Ti analytical rotor. The protein signal was detected by continuously monitoring the absorbance at 280 nm during high-speed centrifugation at 42,000 rpm for 4 hours at 20 °C, with a time interval of 480 seconds and a step size of 0.002 cm. Numerous scans of the sedimentation velocity data were collected and analyzed globally with SEDFIT software^41^,^42^. All size distributions were determined with a confidence level of *p* = 0.95, a best-fit average anhydrous frictional ratio (*f/f*_0_), and a resolution of 200 sedimentation coefficients between 0.1 and 20.0 S.

### Determination of the Dissociation Constant of Dimeric PAD4

The dissociation constant (*K*_d_) of dimeric PAD4 was analyzed using the monomer-dimer equilibrium model in the SEDPHAT program^43^,^44^. The sedimentation velocity data collected at three different enzyme concentrations were globally fit with SEDPHAT to obtain the *K*_d_ values of WT and mutant PAD4.

### Monitoring of Thermal Stability by Circular Dichroism Spectrometry

The thermal denaturation of PAD4 and of the mutant enzymes was analyzed by circular dichroism (CD) spectrometry using a Jasco J-815 spectropolarimeter (JASCO Research Ltd., Canada). A protein sample (0.5 mg/ml) in 30 mM Tris-HCl (pH 7.4) was loaded into a 0.1-cm quartz cuvette and scanned from 250 nm to 190 nm. The ellipticity of all samples was recorded at 222 nm to analyze the conformational changes in the protein throughout the thermal denaturation process. The mean residue ellipticity (θ) at 222 nm was calculated using Equation 2:


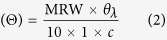


where MRW is the mean residue weight, 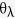 is the measured ellipticity in degrees at wavelength λ, l is the cuvette pathlength (0.1 cm), and *c* is the protein concentration in g/ml.

To determine the melting temperature (*T*_m_) of thermal denaturation, the unfolding curves of the denaturation process were assumed to follow a two-state unfolding mechanism and were fit using the following equations described by Yadav^45^ and Campos^46^.

For a two-state (native and unfolded state) model, *T*_m_ can be calculated by Equation 3:


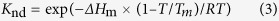


where *K*_nd_ is the equilibrium constant for the denaturation process, *ΔH*_m_ is the van’t Hoff enthalpy at the temperature *T*_m_, T is the absolute temperature in Kelvin, and R is the gas constant.

*K*_nd_ can be calculated using Equation 4:


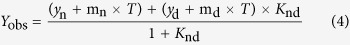


where *Y*_obs_ represents the observed signal, *Y*_n_ and *Y*_d_ represent the signals of the native and denatured states during denaturation, respectively, and *m*_n_ and *m*_d_ are the native and denatured baseline slopes, respectively.

### Molecular Dynamics (MD) Simulation

The initial structure of PAD4 was based on the crystal structure (PDB ID: 1WDA) published in 2004^33^. We used MODELLER^47^ to add the missing residues to the crystal structure and to build the structures of the two mutants W548A and W548F. MD simulations of the WT and mutant proteins were performed using GROMACS version 4.6.7^48^ with the charmm36 force field. For each simulation run, the initial monomeric structure was soaked in an orthorhombic water box, and the net charge was neutralized by the addition of sodium or chloride ions (at 150 mM salt concentration). Long-range electrostatics were addressed using the particle mesh Ewald method. A possible bad geometry from the initial structure was removed using the steepest descent energy minimization until energy convergence reached 1,000 kJ/(mol·nm). The system was then subjected to equilibration at 300 K and normal constant pressure (1 bar) for 100 ps under the conditions of position restraints for heavy atoms and LINCS constraints. The equilibrated structure was used to perform the 20-ns production run at 300 K. The virtual site hydrogen was used to remove the angle vibrations involving hydrogens and to speed up the calculations. The time step of the simulation was set to 5 fs, and the coordinates were saved for analysis every 100 ps. The last 10-ns trajectories were analyzed using GROMACS utilities. The final structures were visualized with PyMOL^49^.

## Additional Information

**How to cite this article**: Lee, C.-Y. *et al*. Molecular Interplay between the Dimer Interface and Substrate-Binding Site of Human Peptidylarginine Deiminase 4. *Sci. Rep.* **7**, 42662; doi: 10.1038/srep42662 (2017).

**Publisher's note:** Springer Nature remains neutral with regard to jurisdictional claims in published maps and institutional affiliations.

## Supplementary Material

Supplementary Files

## Figures and Tables

**Table 1:** Kinetic and biophysical parameters of the human PAD4 substrate-binding-site mutants.

PAD4	*K*_m,BAEE_ (mM)	*k*_cat_ (s^−1^)	*k*_cat_/*K*_m,BAEE_ (s^−1^ mM^−1^)	^1^*T*_m_ (°C)	^2^Quaternary structure	^3^*K*_d_ (μM)
WT	0.5 ± 0.04	11.7 ± 0.30	23.4	51	D	0.10 ± 0.001
R372K	N.D.	N.D.	N.D.	—	—	—
R372A	3.7 ± 0.40	0.07 ± 0.003	0.02	—	—	—
R372Q	2.3 ± 0.37	0.12 ± 0.01	0.05	—	—	—
R374A	0.7 ± 0.08	7.8 ± 0.20	11.1	—	—	—
R374Q	0.4 ± 0.05	3.7 ± 0.08	9.3	—	—	—
R639A	0.7 ± 0.08	5.0 ± 0.13	7.1	—	—	—
W347A	N.D.	N.D.	N.D.	47	D	1.02 ± 0.01
W347F	N.D.	N.D.	N.D.	—	—	—
V469A	5.3 ± 0.77	0.8 ± 0.04	0.15	50	D	2.13 ± 0.02
V469L	1.0 ± 0.19	0.1 ± 0.003	0.1	—	—	—
V469T	0.1 ± 0.03	0.04 ± 0.001	0.4	—	—	—

^1^The *T*_m_ values are derived from a two-state model that was used to fit the thermal denaturation curves detected by CD (Figure S1).

^2^The quaternary structure of PAD4 was determined by AUC; D indicates dimer.

^3^The *K*_d_ value was obtained by fitting the global data for sedimentation velocity using three different PAD4 concentrations (Fig. 2).

N.D. indicates that no enzyme activity was detectable.

**Table 2:** Kinetic and biophysical parameters of the human PAD4 dimer-interface mutants.

PAD4	*K*_m,BAEE_ (mM)	*k*_cat_ (s^−1^)	*k*_cat_/*K*_m,BAEE_ (s^−1^ mM^−1^)	^1^*T*_m_ (°C)	^2^Quaternary structure	^3^*K*_d_ (μM)
WT	0.5 ± 0.04	11.7 ± 0.30	23.4	51	D	0.10 ± 0.001
L6A	0.6 ± 0.06	7.3 ± 0.24	12.2	44	M/D	7.60 ± 0.060
L6I	0.5 ± 0.03	10.9 ± 0.12	21.8	45	D	2.13 ± 0.028
L6D	0.5 ± 0.07	4.5 ± 0.14	9.0	43	M	24.7 ± 0.26
L279A	0.4 ± 0.02	5.5 ± 0.10	13.8	46	M/D	5.97 ± 0.08
L279I	0.4 ± 0.03	11.2 ± 0.15	28	48	D	0.33 ± 0.003
L279D	1.9 ± 0.22	3.6 ± 0.06	1.9	45	M	65.3 ± 0.47
V283A	0.7 ± 0.09	8.0 ± 0.41	11.4	47	M/D	9.25 ± 0.08
V283T	0.9 ± 0.09	5.5 ± 0.14	6.3	55	M/D	8.91 ± 0.06
V283I	0.5 ± 0.05	9.5 ± 0.33	19	54	D	0.18 ± 0.001
V283D	N.D.	N.D.	N.D.	46	M	46.7 ± 0.35
V284A	1.5 ± 0.42	5.5 ± 0.01	3.7	47	D	2.40 ± 0.02
V284I	0.5 ± 0.03	10.9 ± 0.24	21.8	52	D	0.18 ± 0.001
V284D	0.9 ± 0.09	2.9 ± 0.08	3.2	47	D	2.59 ± 0.02
F285A	0.8 ± 0.05	6.9 ± 0.17	8.6	47	D	0.75 ± 0.01
F541A	0.7 ± 0.05	7.7 ± 0.20	11	48	D	0.25 ± 0.003
W548A	N.D.	N.D.	N.D.	46	M	88.7 ± 0.73
W548F	1.4 ± 0.25	6.2 ± 0.46	4.6	47	M/D	12.9 ± 0.08
W548K	0.3 ± 0.07	0.6 ± 0.05	2	46	M	84.5 ± 0.54
F576A	0.4 ± 0.02	9.8 ± 0.13	24.5	48	D	0.12 ± 0.001

^1^The *T*_m_ values are derived from a two-state model that was used to fit the thermal denaturation curves detected by CD (Figure S1).

^2^The quaternary structure of PAD4 was determined by AUC; M indicates monomer, and D indicates dimer.

^3^The *K*_d_ value was obtained by fitting the global data for sedimentation velocity using three different PAD4 concentrations (Figs 3, 4 and 5 and Figure S2).

N.D. indicates that no enzyme activity was detectable.

**Table 3:** Kinetic and biophysical parameters of the human PAD4 I-loop and S-loop mutants.

PAD4	*K*_m,BAEE_ (mM)	*k*_cat_ (s^−1^)	*k*_cat_/*K*_m,BAEE_ (s^−1^ mM^−1^)	^1^*T*_m_ (°C)	^2^Quaternary structure	^3^*K*_d_ (μM)
WT	0.5 ± 0.04	11.7 ± 0.30	23.4	51	D	0.10 ± 0.001
C434A	0.4 ± 0.01	8.0 ± 0.08	20	50	D	0.36 ± 0.01
Y435A	3.2 ± 1.09	2.8 ± 0.39	0.9	47	M	22.0 ± 0.24
R441A	1.7 ± 0.41	2.3 ± 0.22	1.4	47	M/D	14.23 ± 0.16
D465A	0.4 ± 0.03	3.5 ± 0.10	8.8	46	M/D	8.30 ± 0.11

^1^The *T*_m_ values are derived from a two-state model that was used to fit the thermal denaturation curves detected by CD (Figure S1).

^2^The quaternary structure of PAD4 was determined by AUC; M indicates monomer, and D indicates dimer.

^3^The *K*_d_ value was obtained by fitting the global data for sedimentation velocity using three different PAD4 concentrations (Fig. 7).
