# Supplementary material for: Molecular Interplay between the Dimer Interface and the Substrate-Binding Site of Human Peptidylarginine Deiminase 4
Source: Sci Rep. 2017 Feb 17;7:42662. doi: 10.1038/srep42662 (PMC5314407; doi:10.1038/srep42662)
Supplement: Supplementary Files [file srep42662-s1.pdf]

Molecular Interplay between the Dimer Interface and Substrate-Binding Site of Human Peptidylarginine Deiminase 4

Chien-Yun Lee, Chu-Cheng Lin, Yi-Liang Liu, Guang-Yaw Liu, Jyung-Hurng Liu, and Hui-Chih Hung

Table S1: The sequences of the mutagenic primers

**PAD4_L6A**	5'-GGCCCAGGGGACAGCCATCCGTGTGACCCCAG-3'
**PAD4_L6I**	5'-CATGGCCCAGGGGACAATCATCCGTGTGACCCCAG-3'
**PAD4_L6D**	5'-GCATCATGGCCCAGGGGACAGACATCCGTGTGACCCCAGAGC-3'
**PAD4_L279A**	5'-CTGGACACGTCCAACCTGGAGGCCCCCGAGGCTGTGGTGTTC-3'
**PAD4_L279I**	5'-GACACGTCCAACCTGGAGATCCCCGAGGCTGTGGTGTTC-3'
**PAD4_L279D**	5'-GACACGTCCAACCTGGAGGACCCCGAGGCTGTGGTGTTC-3'
**PAD4_V283A**	5'-GCTCCCCGAGGCTGCGGTGTTCCAAGACAG-3'
**PAD4_V283I**	5'-GAGCTCCCCGAGGCTATAGTGTTCCAAGACAGC-3'
**PAD4_V283D**	5'-GGAGCTCCCCGAGGCTGATGTGTTCCAAGACAGC-3'
**PAD4_V284A**	5'-CTCCCCGAGGCTGTGGCGTTCCAAGACAGCGTG-3
**PAD4_V284I**	5'-CTCCCCGAGGCTGTGATATTCCAAGACAGCGTG-3'
**PAD4_V284D**	5'-GCTCCCCGAGGCTGTGGATTTCCAAGACAGCGTGG-3'
**PAD4_F285A**	5'-CCGAGGCTGTGGTGGCCCAAGACAGCGTGG-3'
**PAD4_W347A**	5'-GAACATGGATGACCAGGCGATGCAGGATGAAATGG-3'
**PAD4_W347F**	5'-GAACATGGATGACCAGTTCATGCAGGATGAAATGG-3'
**PAD4_R372A**	5'-GGTCTTCGACTCTCCAGCGAACAGAGGCCTGAAG-3'
**PAD4_R372K**	5'-GGTCTTCGACTCTCCAAAAAACAGAGGCCTGAAG-3'
**PAD4_R372Q**	5'-GGTCTTCGACTCTCCACAGAACAGAGGCCTGAAG-3'
**PAD4_R374A**	5'-CGACTCTCCAAGGAACGCGGGCCTGAAGGAGTTTC-3'
**PAD4_R374Q**	5'-CGACTCTCCAAGGAACCAGGGCCTGAAGGAGTTTC-3'
**PAD4_C434A**	5'-GATTCTCTTCGGGGACAGCGCTTATCCCAGCAATGACAG-3'
**PAD4_Y435A**	5'-GTCATTGCTGGGAGCACAGCTGTCCCCGAAGAGAATC-3'
**PAD4_R441A**	5'-CCCTGGTGCATCTGCGCGCTGTCATTGCTGG-3'
**PAD4_D465A**	5'-GTGAAGCTCTATTCTGCCTGGCTGTCCGTGGG-3'
**PAD4_V469A**	5'-CTGGCTGTCCGCGGGCCACGTGG-3'
**PAD4_V469L**	5'-CTGGCTGTCCCTGGGCCACGTGG-3'
**PAD4_V469T**	5'-CTGGCTGTCCACCGGCCACGTGG-3'
**PAD4_F541A**	5'-CATTGAGAGAACATAATTCAGCCGTGGAGAGATGCATCGAC-3'
**PAD4_W548A**	5'-GAGAGATGCATCGACGCGAACCGCGAGCTGCT-3'
**PAD4_W548F**	5'-GAGAGATGCATCGACTTCAACCGCGAGCTGCTGA-3'
**PAD4_W548K**	5'-GGAGAGATGCATCGACAAGAACCGCGAGCTGCTG-3'
**PAD4_F576A**	5'-CCGCAGCTCTTCAAGCTCAAAGAGGCCTCTAAGGCGGAAGCTTTTTTC-3'
**PAD4_R639A**	5'-CACCTACCACATCGCGCATGGGGAGGTGC-3'

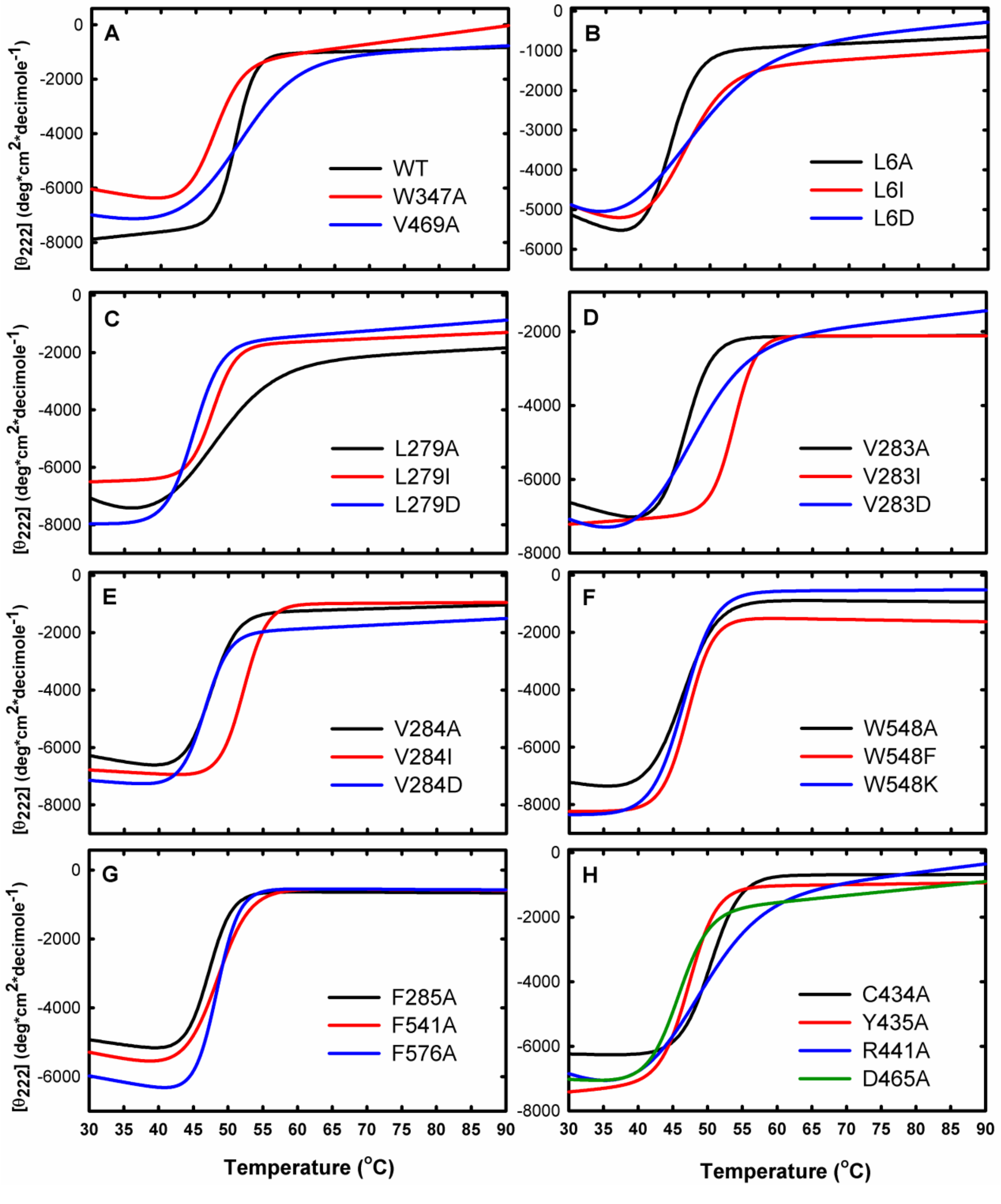

Figure S1. Thermal stability of human PAD4 WT and its mutants. Thermal denaturation of PAD4 was monitored by CD. The experimental data are presented as the mean residue ellipticity at 222 nm (θ_{222}). All data were fit with a two-state model, and the fitting results are shown as a solid line.

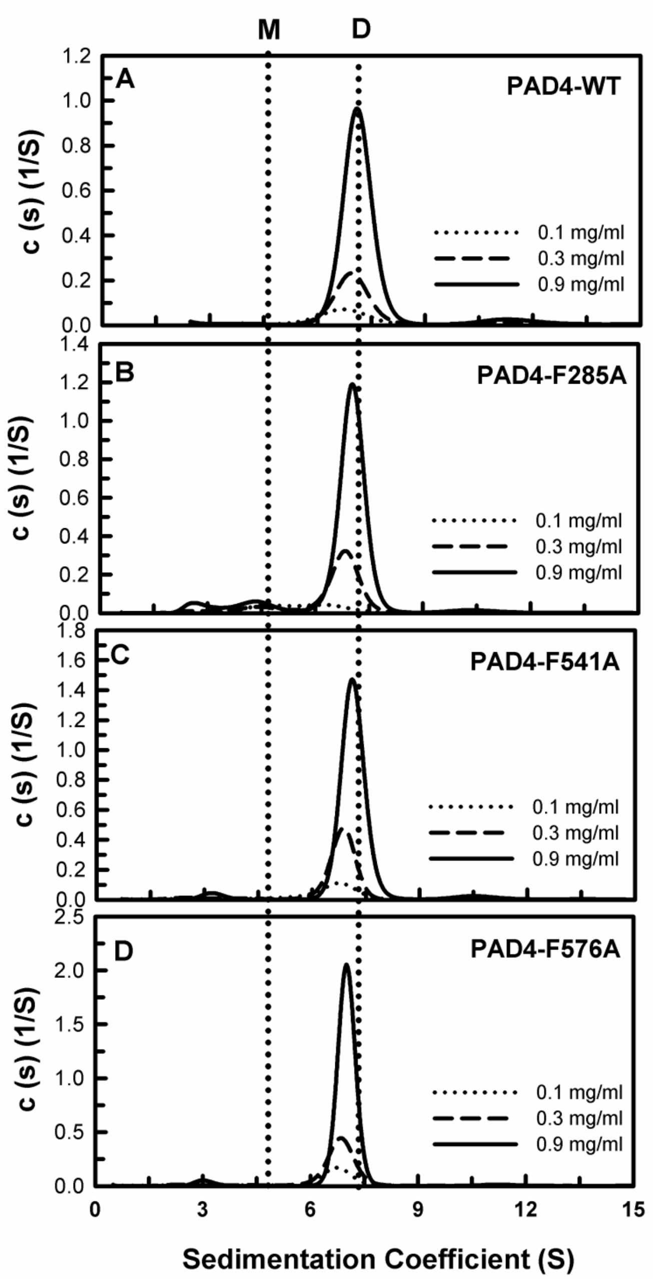

Figure S2. Continuous sedimentation coefficient distributions of PAD4 WT and the other dimer interface mutants. A. WT; **B.** F285A; **C.** F541A; **D.** F576A. The K_d values of these mutants are shown in Table 2. M, monomer; D, dimer.

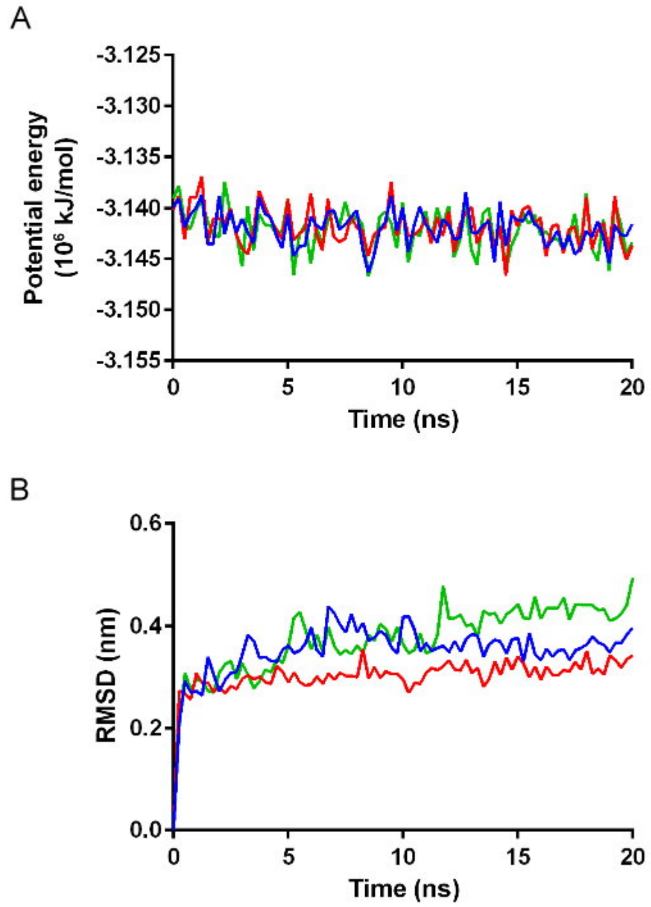

Figure S3. Analysis of the MD trajectories generated by GROMACS. The trajectories for **A.** the potential energy and **B.** RMSD of the backbone atoms are shown. The blue, green, and red lines indicate the WT, W548A, and W548F mutant structures, respectively.

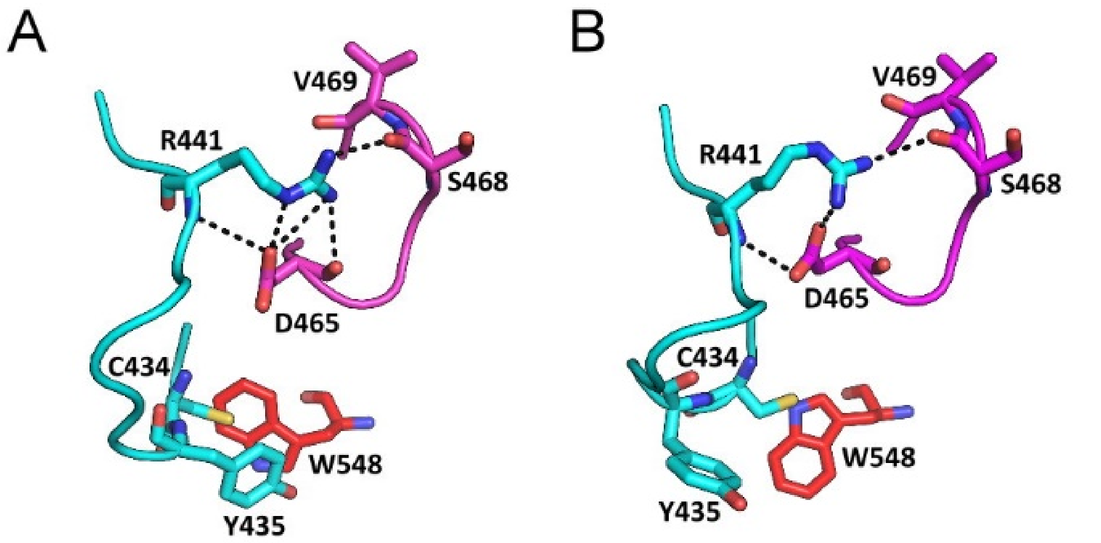

Figure S4. Comparison of the loop conformations in the MD and crystal structures of PAD4. **A.** MD structure; **B.** crystal structure (PDB ID: 1WDA)**.** I-loop and S-loop are colored in cyan and magenta, respectively. The important residues are highlighted as stick models and labeled. The possible polar contacts are represented as black dashed lines.

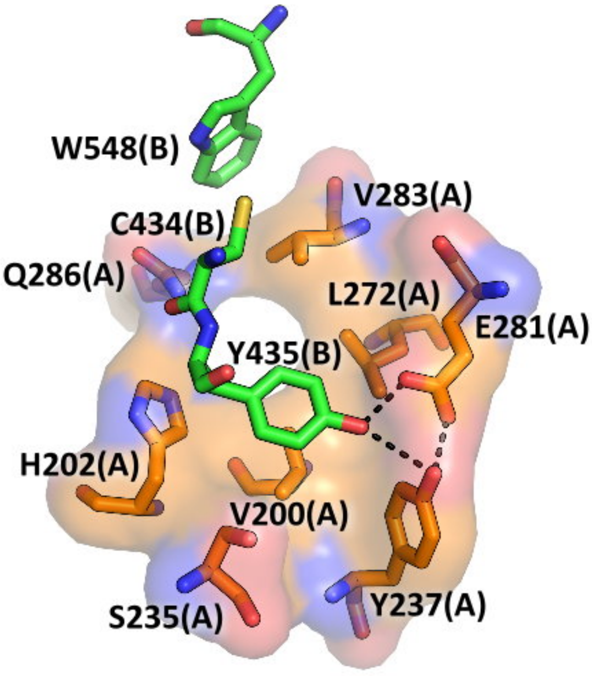

Figure S5. The interaction between I-loop and dimer interface in the crystal structure of PAD4 (PDB ID: 1WDA). The green and orange sticks indicate the residues from the different subunits, respectively. The possible polar contacts are represented as black dashed lines.
